# Dimension dependence of current injection path in GaInN/GaN multi-quantum-shell (MQS) nanowire-based light-emitting diode arrays

**DOI:** 10.1515/nanoph-2023-0051

**Published:** 2023-06-16

**Authors:** Sae Katsuro, Weifang Lu, Kazuma Ito, Nanami Nakayama, Soma Inaba, Ayaka Shima, Shiori Yamamura, Yukimi Jinno, Naoki Sone, Kai Huang, Motoaki Iwaya, Tetsuya Takeuchi, Satoshi Kamiyama

**Affiliations:** Department of Materials Science and Engineering, Meijo University, 1-501 Shiogamaguchi, Tenpaku-ku, Nagoya, 468-8502, Japan; Fujian Key Laboratory of Semiconductor Materials and Applications, CI Center for OSED, Department of Physics, Xiamen University, Xiamen 361005, China; Future Display Institute in Xiamen, Xiamen 361005, China

**Keywords:** current injection path, electroluminescence (EL), ITO area, micro-LED, MQS nanowire, p-electrode area

## Abstract

To light emitting diodes (LEDs), solving the common non-uniform current injection and efficiency degradation issues in (0001) plane micro-LED is essential. Herein, we investigated the light emission characteristics of various mesa sizes and different p-electrode areas toward the realization of coaxial GaInN/GaN multi-quantum-shell (MQS) nanowires (NWs)-based micro-LEDs. As the mesa area was reduced, the current leakage decreases, and further reduction of the area showed a possibility of realizing micro-LED with less current leakage. The large leakage path is mainly associated with the defective MQS structure on the (0001) plane area of each NW. Therefore, more NWs involved in an LED chip will induce higher reverse leakage. The current density-light output density characteristics showed considerably increased electroluminescence (EL) intensity as the mesa area decreased, owing to the promoted current injection into the efficient NW sidewalls under high current density. The samples with a mesa area of 50 × 50 µm^2^ showed 1.68 times higher light output density than an area of 100 × 100 µm^2^ under a current density of 1000 A/cm^2^. In particular, the emission from (1-101) and (10-10) planes did not exhibit an apparent peak shift caused by the quantum-confined Stark effect. Furthermore, by enlarging the p-electrode area, current can be uniformly injected into the entire chip with a trade-off of effective injection to the sidewall of each NW. High performance of the MQS NW-based micro-LED can be expected because of the mitigated efficiency degradation with a reducing mesa area and an optimal dimension of p-electrode.

## 1 Introduction

Under the constant innovation of display technology, group III nitride semiconductors are deemed as promising materials for the micro-light-emitting diodes (micro-LEDs) [1, 2]. Because of the high peak brightness, low power consumption, high speed, and cost reduction, micro-LEDs are expected to apply to smartwatches, smartphones, and three-dimensional augmented and virtual reality displays [1, 2]. In planar LEDs, the polar (0001) plane-based active structures usually contain piezoelectric field-induced quantum-confined Stark effect (QCSE), which causes a decrease in luminous efficiency, especially in the long wavelength range [3], [4], [5]. So far, various efforts have been made toward the realization of micro-LED with different emission wavelengths [6–10], while the degradation issues in external quantum efficiency (EQE) with a decreasing LED chip size were also widely reported [11, 12]. As the device size decreases, the area of the edge with respect to the total emission area increases, leading to a raised ratio of the nonradiative recombination centers at the edge surface to the device area [13–16]. Even though the dry etching-induced surface damages can be partially passivated by atomic layer deposited dielectric films, the recovery is still limited to improve the emission efficiency [17]. In addition, the intrinsic influence of QCSE can cause thermal loss of carriers before they reach the multiple quantum well regions in LEDs, increasing the nonradiative recombination in the p-GaN region, and reducing the EQE at small chip sizes [6, 18, 19]. Furthermore, blue shifts of 9 nm for blue micro-LEDs and 15 nm for green micro-LEDs occur with increasing current density from 1 A/cm^2^–1 kA/cm^2^. The emission wavelength is not constant depending on the chip size and varies within a deviation range of 5 nm [20, 21]. Overall, one of the key points to improve the emission efficiency and wavelength stability in micro-LEDs is to suppress the nonradiative recombination centers in sidewalls and reduce the effect of QCSE from (0001) plane active structures.

GaInN/GaN-based multi-quantum shell (MQS) nanowire (NW) is of great interest as a promising material that can alleviate the issues mentioned above [22]. The MQS active structures are coaxially grown on the semipolar (1-101) plane and nonpolar (10-10) planes surface of the NWs, which can effectively reduce the influence of the piezoelectric field. As a result, there is a possibility for suppressing QCSE, improving luminous efficiency, solving the green gap problem, and realizing a high-efficiency micro-LED [23–27]. Compared with the planar (0001) plane LEDs, the active layer in NW LED is featured with a three-dimensional structure, increasing the luminous area and light extraction efficiency [28, 29]. In addition, there are many advantages, such as controlling the emission wavelength and obtaining low dislocation crystals by reducing the diameter of the NWs [30–32]. Moreover, the (1-101) planes are easier to incorporate in In than the (10-10) plane owing to their spatial growth characteristics, and by taking advantage of the (1-101) plane, MQS NW LEDs are also promising for realizing highly efficient LEDs in long wavelength range [33–35]. Specifically, the MQS NWs likewise show prospective application in micro-LEDs, because the surface recombination rate at the mesa edge is lower than that of the planar micro-LEDs owing to the minimal exposed region of the active region after mesa fabrication. Therefore, MQS NW is a promising candidate to suppress the enhancement of nonradiative recombination near the mesa edge region of planar micro-LEDs. Since the emission mechanism related to the dimension of devices is still unclear in MQS-NW LEDs, it is particularly essential to gain insight into the electrical and optical characteristics of the chip size and to identify the related issues. Therefore, as an initial step toward realizing NW-based micro-LED, we investigated the dependence of the current injection paths and corresponding luminescence characteristics on the device size.

As a preliminary investigation toward the realization of GaInN/GaN MQS NW-based full-color micro-LED, we systematically compared the emission properties of NW LED with different emission areas. Eight types of NW LEDs with different mesa and p-electrode areas were fabricated on the NW samples grown under identical conditions. The current injection path and emission characteristics were evaluated and discussed from the viewpoints of current–voltage curves, current–light output characteristics, and electroluminescence (EL) spectra. The results of different p-electrodes indicated a trade-off between the current diffusion through all the NWs and effective injection to the sidewall of each NW in the LED devices.

## 2 Experimental section

**Figure 1:**
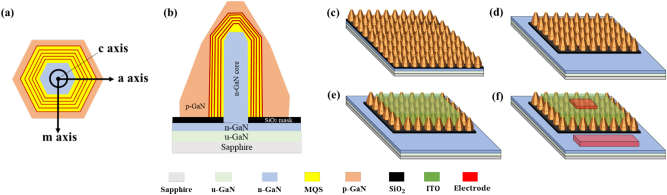
Schematic of the structures and device fabrication for nanowire (NW)-based light-emitting diodes (LEDs): (a) cross-sectional view from the *c*-axis direction of the NW; (b) cross-section details from the *m*-axis direction of the NW; (c) NWs with activation inside the MOCVD chamber after growth; (d) removal of NWs in the n-electrode area and exposure of n-GaN layer by dry etching; (e) indium tin oxide (ITO) sputtering and thermal annealing; (f) deposition of Cr/Ni/Au films on the p- and n-electrodes.

The NW samples used in the LED process were prepared by metalorganic vapor phase epitaxy (MOVPE) using the selective area growth method [36, 37]. First, a 30 nm thick SiO_2_ mask layer was deposited on the n-GaN/sapphire substrate using a radiofrequency magnetron sputtering system (CFS-4EP, Shibaura Mechatronics Co., Yokohama City, Kanagawa, Japan). After that, a hole pattern of a triangular lattice array with a diameter of 300 nm and a pitch of 1200 nm was formed by nanoimprint lithography, and the GaN inside the holes was exposed by inductively coupled plasma (ICP) etching (MV06-7001-0, ULVAC, Inc., Chigasaki City, Kanagawa Japan). Then, n-GaN core NW, five pairs of GaInN/GaN MQS with AlGaN spacers, and p-GaN shell were sequentially grown by MOVPE, as illustrated in Figure 1(a) and (b). Approximately 800 nm-tall Si-doped GaN core NWs were grown at a high temperature of 1140 °C for 70 s. The detailed growth conditions for the MQS structures can be found in our previous work [23, 24, 38], [39], [40]. Regarding the p-GaN shell, an Mg-doped (∼3 × 10^19^ cm^−3^) p-GaN was grown at 930 °C for 6 min, followed by a thin shell with higher Mg doping (∼6 × 10^19^ cm^−3^) concentration. The Mg/Ga ratios in these two p-GaN shells were set to 5.45 × 10^−3^ and 1.09 × 10^−2^, respectively, by controlling the supplied flow rates of trimethylgallium (TMG) and bis-cyclopentadienyl magnesium (Cp_2_Mg) precursors. In conventional fabrication, activation for the p-GaN shells is generally performed via rapid thermal annealing (RTA) processing to remove the hydrogen contained in p-GaN and reduce the resistance. However, without nitrogen purification in the RTA system, the thermal activation may also introduce an oxide film on the surface of the p-GaN shell under ambient nitrogen and oxygen conditions, which can cause the degradation of the Ohmic contact formation. Therefore, in this work, the activation annealing of all the NW samples was performed inside the MOVPE reactor at 650 °C for 30 min in ambient N_2_, i.e., an oxygen-free atmosphere.

Afterward, eight different types of LED devices with varied shapes and sizes of the mesa and electrode areas were fabricated on the samples. The detailed dimensions of mesa and p-electrodes in the devices are shown in Table 1. In samples b, c, d, and e, the indium tin oxide (ITO) region was designed to be same shape (200 × 100 μm^2^), while the p-electrode area was decreased. To investigate the effect of emission region, only the ITO area was changed in samples d, g, and h. The distinctive shape of samples a and f was referred to the design that was reported in our previous work [41, 42]. Although the ITO area is not rectangular, the surface areas of samples a and f are equal to be 340 × 340 and 120 × 120 μm^2^, respectively. Figure 1(c)–(f) show the fabrication process flow of NW-based LED devices, including removing NWs around the mesa area, ITO deposition, and electrode formation. To remove the NWs around the mesa and n-electrode parts, the Ni film with a thickness of 150 nm was vapor-deposited on the samples by electron beam (EB) deposition (MA08-3065, ULVAC, Inc., Chigasaki City, Kanagawa, Japan) except for the n-electrode part. After coating a resist on the entire samples, the ICP etching with a mixed gas of Cl_2_ and Ar was performed to selectively etch off the NWs in the designated area. Subsequently, the transparent current spreading layer of ITO was deposited using a radio frequency magnetron sputtering system. The ITO layer with 60 nm thickness was deposited under the Ar and O_2_ gas mixed atmosphere, with RF power of 20 and 100 W sequentially in two steps. To reduce the resistance in ITO, thermal annealing was performed at 600 °C for 4 min in N_2_ atmosphere using rapid thermal processing (RTA) (MR094017-0, ULVACRIKO, INC., Tsuzuki City, Yokohama, Japan). Finally, Cr, Ni, and Au layers with thicknesses of 10, 20, and 200 nm, respectively, were deposited as the p- and n-electrode area by EB evaporation. The structural morphologies of the NW were observed with a scanning transmission electron microscope (SEM) (SU70, Hitachi High-Technologies Co., Minato City, Tokyo, Japan) under an acceleration voltage of 3.0 kV. The optical characteristics of NW LEDs were evaluated by EL measurements via a fiber spectrometer (USB4000, Ocean Optics Co., United States) and current–voltage–light output (I–V–L) characterizations (4156c, Agilent Technology, Santa Clara CA, USA). In both cases, the detection was carried out from the back side of the substrate, while the position of the detector was fixed for all the samples.

**Table 1:** Chip size of ITO area and p-electrode area.

Sample name	A	b	c	d	e	f	g	h
ITO area	340 × 340	200 × 100	200 × 100	200 × 100	200 × 100	120 × 120	100 × 100	50 × 50
S (µm^2^)	115,600	20,000	20,000	20,000	20,000	14,400	10,000	2500
p-electrode area (µm^2^)	24,000	190 × 40	180 × 30	40 × 40	30 × 30	130 × 30	40 × 40	40 × 40
Ratio of p-electrode to ITO area	0.208	0.38	0.27	0.08	0.045	0.27	0.16	0.64

## 3 Results and discussion

### 3.1 Morphology inspection of the NW structures during the process

**Figure 2:**
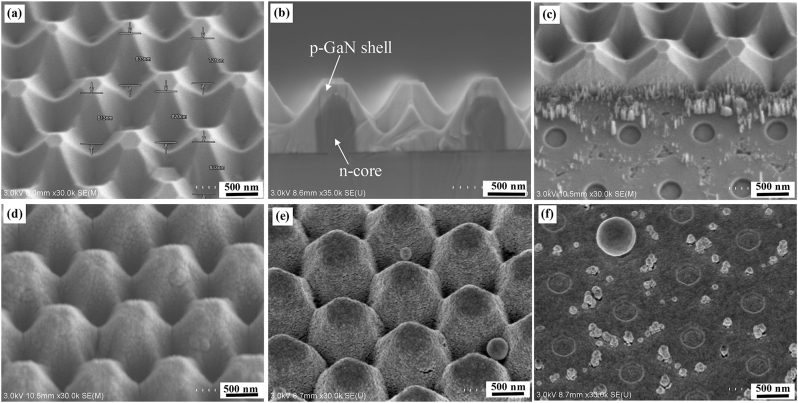
SEM characterizations of the NWs during fabrication process. (a) Tilted and (b) cross-sectional SEM images of the NWs after MOVPE growth. (c) SEM image of the region between n-electrode and NWs after etching. Tilted-view SEM image of the NWs (d) after ITO sputtering and (e) metallic film deposition on the p-electrodes. (f) SEM image of the n-electrode surface after deposition of Cr/Ni/Au films.

**Figure 3:**
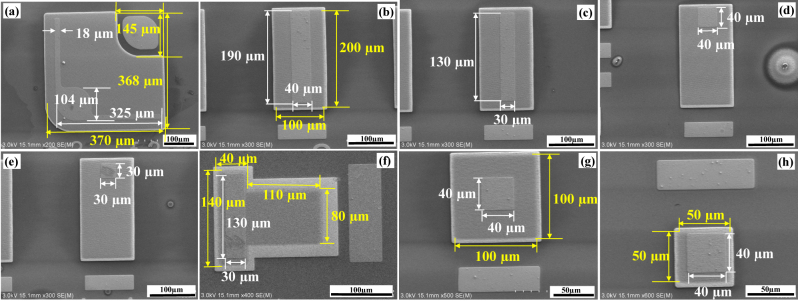
SEM images of the fabricated NW LEDs. Details of the chip layout for (a) sample a; (b) sample b; (c) sample c; (d) sample d; (e) sample e; (f) sample f; (g) sample g; and (h) sample h (white line: length of p-electrode; yellow line: length of ITO region).

During the fabrication, the surface morphology of the NWs was inspected by SEM measurements, as shown in Figure 2(a)–(f). After MOVPE growth, the p-GaN shell was connected, forming a ridge between the NWs along the junction. The hexagonal shape of the (0001)-plane on the tip area was also clearly visible. The cross-sectional SEM image in Figure 2(b) shows that the p-GaN shell was uniformly grown on the whole NWs. Furthermore, the color contrast in Figure 2(b) confirms that the heights of the n-GaN core and the entire NW grown with the p-GaN shell are 800 and 990 nm, respectively. To promote the conformal deposition of the ITO layer, the height of the n-core NW was controlled, and the p-GaN growth was conducted based on the optimized conditions [23, 43–45]. Because the annealing temperature in the MOCVD chamber was lower than that of the p-GaN shell growth, it is unlikely that the morphology was deformed during annealing in the chamber under ambient N_2_. The surface features of all the samples in this work were confirmed to be the same owing to the simultaneous growth and annealing processes. Figure 2(c) shows the tilted-view SEM image of the region between n-electrode and NWs after etching. The observed holes for n-GaN core growth indicate that the n-GaN substrate under the NWs was fully exposed. Nevertheless, a few residual p-GaN spikes existed close to the un-etched NWs because of the edge effect by the overlap between the Ni mask in the mesa and resist in the n-electrode region. Afterward, ITO was uniformly coated on the NWs in the mesa region, as confirmed in Figure 2(d). Figure 2(e) and (f) show the surface morphologies of the p- and n-electrodes, respectively, after the Cr/Ni/Au film deposition. The dimensional details of the fabricated NW LED chips in samples a–h were confirmed by the SEM inspection, as shown in Figure 3(a)–(h), respectively. Sizes of the mesa and electrode regions were reliable as desired after the processes. The ratios of p-electrode to the ITO region for samples a–h were calculated to be 0.208, 0.38, 0.27, 0.08, 0.045, 0.27, 0.16, and 0.64, respectively. The samples a, d, f, g, and h with different mesa areas were compared to analyze the effect of the total emission region, despite the electrode size variation. For samples b, c, d, and e, the shape (200 × 100 µm^2^) of the mesa region was kept constant, while the sizes of p-electrodes varied as 190 × 40, 180 × 30, 40 × 40, and 30 × 30 µm^2^, respectively.

### 3.2 Evaluation of optical characteristics based on the difference in the mesa area

**Figure 4:**
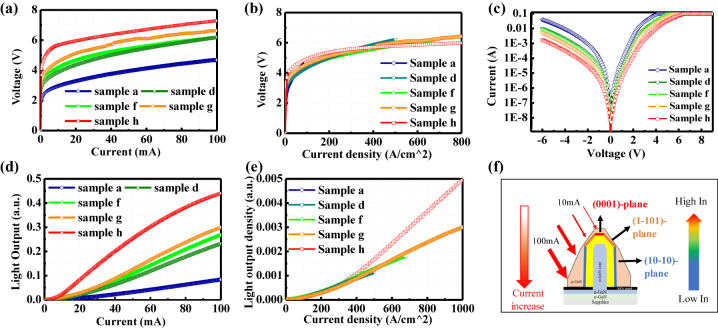
*I*–*V*–*L* curves and schematic images of the current injection paths in the NW samples. (a) *I*–*V* characteristics in samples a, d, f, g, and h with different ITO mesa areas. (b) The voltage was replotted as a function of current density. (c) The corresponding semi-log *V*–*I* characteristics measured from −6 to 9 V. (d) The *I*–*L* characteristics in samples a, d, f, g, and h. (e) The light output was replotted as a function of current density. (f) The schematic diagram of the emission surface changed depending on the current injection value and the fraction of InN.

To evaluate the optical characteristics depending on the difference in the mesa area, reduced ITO regions with 340 × 340, 200 × 100, 120 × 120, 100 × 100, and 50 × 50 μm^2^ were prepared in samples a, d, f, g, and h, respectively. Because the size and the shape of the NW LEDs are different in these samples, the corresponding p-electrodes were tuned for the benefit of the current injection. From the *I*–*V* curves in Figure 4(a), the threshold voltage and overall resistance increase as the ITO area decreases. Because the total number of NWs successively decreased from sample a to h, more current can be injected into the sidewall of the NWs in sample h under the same injection current. In this case, the high resistance of the thicker p-GaN shell on the sidewall path also became evident in the *I*–*V* curves. Since the p-GaN shell on the sidewall was connected and conformally covered by the ITO layer, the contact resistance between ITO and p-GaN was considered to be uniform along the NWs. To further clarify the *I*–*V* characteristics, the relationship between current and voltage was replotted as a function of the current density, as shown in Figure 4(b). Below the current density of 300 A/cm^2^, more current was injected into the sidewall of the NWs in the LEDs with a smaller mesa area, resulting in a slightly higher operation voltage. Under the same current density beyond 300 A/cm^2^, the difference in operation voltage is relatively small. To inspect the leakage current, the voltage–current curves were measured under reverse and forward bias, as plotted with a semi-log scale in Figure 4(c). The reverse leakage current in the NW LED is relatively high (>10^−3^ A at a reverse voltage of −5 V) and increasing with an enlarged area of the mesa region. The large leakage path was mainly associated with the high-density-dislocations containing in MQS structure on the (0001) plane area of each NW. Because of the high growth rate of MQS on the (0001) plane, the In-content was higher than those on the other planes, and the MQS thickness on the (0001) plane exceeded the critical thickness for 2D growth. Thus, the induced compressive strain easily triggered the formation of screw and Frank-type partial dislocations in the apex p-GaN shell [46, 47]. Therefore, more NWs in the LED will induce higher reverse leakage. Other possible current leakage points include formation of holes in the SiO_2_ mask region and an abnormally grown p-GaN shape need to considered. Nevertheless, improved growth quality of the (0001) plane MQS region or the NWs apex truncated method is expected to alleviate the leakage, as reported in our previous work [24, 48]. Here, the remained (0001) plane MQS region was devoted to analysing the current injection paths along the NWs.

The light output characteristics of samples a, d, f, g, and h are plotted as a function of injection current in Figure 4(d). Under low current injection, the light output intensity is relatively low in all the samples. This is because the injection current was mainly localized at the apex (0001) plane region with relatively low crystallinity. When the current increases, the slope increases owing to the emission from the (1-101) plane and the top of the (10-10) plane of the NWs. A clear enhanced light output was confirmed as the mesa region reduced in the NW LED, especially at the currents above 20 mA. However, the slight saturation in sample h is attributable to the thermal effect with high current injection density. To precisely compare the effect of the ITO mesa area on the emission properties, the light output curves were plotted in terms of current density, as shown in Figure 4(e). For all the five NW LED samples with different mesa areas, the curves inevitably overlap from the current density of 0 to approximately 320 A/cm^2^. It reveals that the emission property under low current injection density coincides with individual NWs among the LED samples regardless of the ITO mesa area. This phenomenon is assignable to the localization of current injection at the apex region under low current density. The light output density of sample h at 1000 A/cm^2^ was approximately 1.68 times higher than that of sample g. The slope is expected to be the same in samples h and g if the current diffusion is the same. However, the ratios of p-electrode to the mesa area in samples g and h are 0.16 and 0.64, respectively. Therefore, it is considered that the current diffusion and the injection efficiency were improved in sample g, resulting in the enhanced light output. In addition, the non-radiative recombination of the edge region, which commonly increases with planar micro-LEDs, is expected to be minimal in NW LEDs, even with reduced mesa area. Figure 4(f) shows the schematic diagram of emission surface change depending on current injection density and spatial distribution of In content. When the current density is high, the current can be injected into the lower part of the NWs. Since the In content decreases from the upper to the lower part, the light emission has a shorter wavelength, and the threshold voltage becomes higher. High performance of the MQS NW-based micro-LED can be expected because of the mitigated efficiency degradation with reducing mesa area.

**Figure 5:**
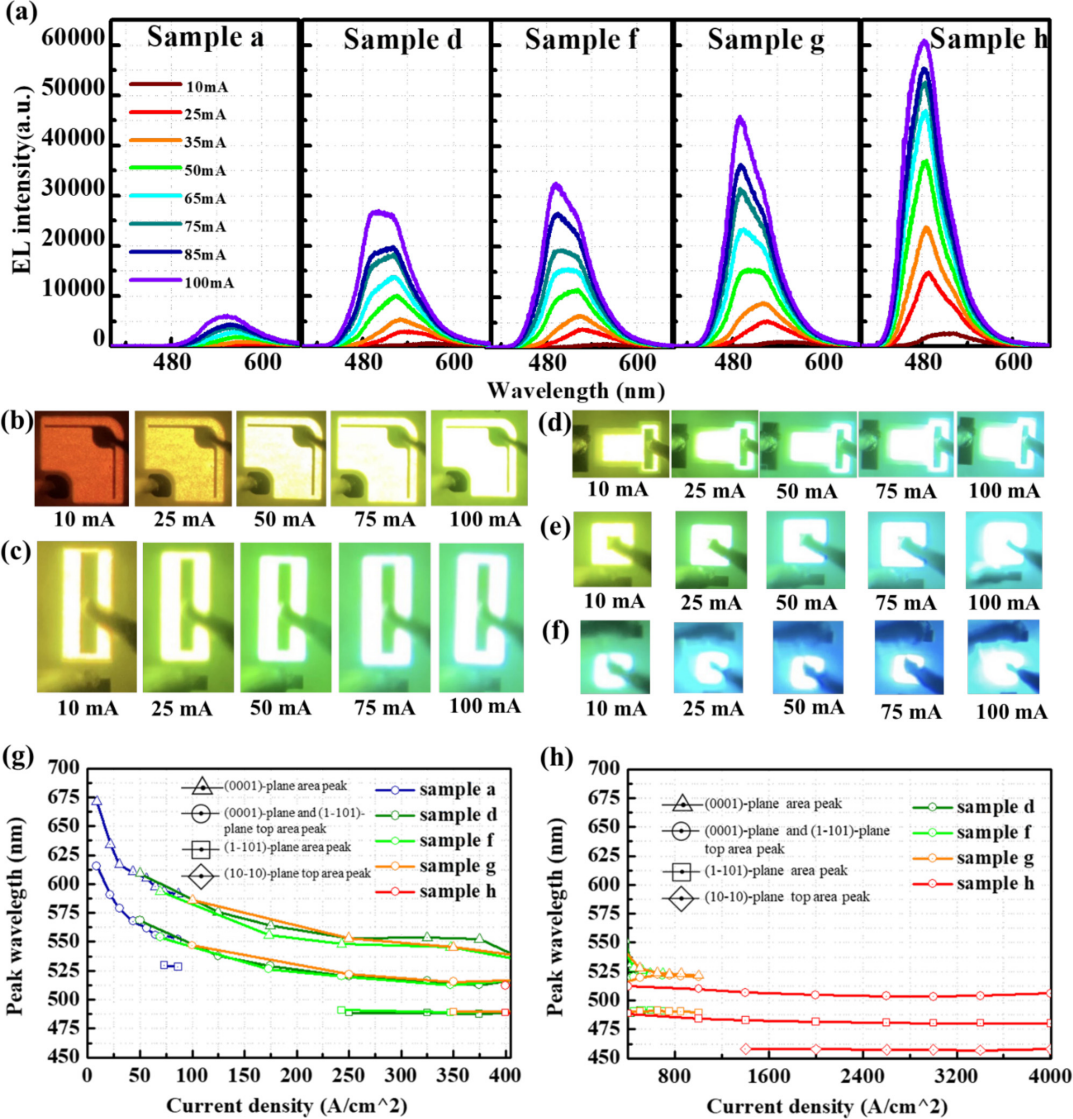
EL spectra and luminescence photos of the samples with different chip sizes. (a) EL spectra of the samples a, d, f, g, and h under different injection currents. Panels (b)–(f) show the luminous photographs of LED chips in samples a, d, f, g, and h, respectively. (g) And (h) show the EL peak wavelength at each current density, separated by the Gaussian fitting. The triangular marks indicate the emission from the (0001) plane at the apex region of the NWs. The circular marks were assignable to the emission from the (0001) plane and upper part of the (1-101) plane regions, while the squares were light emissions from the (1-101) plane. The diamond notation is considered light emission from the upper part of the (10-10) plane.

The EL spectra acquired at different injection currents are plotted in Figure 5(a) for samples a, d, f, g, and h, respectively. The emission peaks of samples a, d, f, g, and h are located at around 550, 500, 492, 491, and 485 nm, respectively. Two emission peaks are observed in samples d and f, especially under current injections above 50 mA. It can be seen that the emission intensity increases as the ITO mesa area of the NW LED decreases, and an enhancement of 12-folds is confirmed as compared between samples a and h. This is because the current density increases as the ITO area decreases, so that more current can reach the lower part of the NWs. As shown in Figure 5(b)–(f), the luminous photographs of the NW LEDs were taken from the front side through the microscope under injection currents of 10, 25, 50, 75, and 100 mA, respectively. For the NW LEDs with a large mesa area in sample a, the emission color was varied from red at 10 mA to bright yellow at 100 mA. The emission color in samples d, f, and g showed a similar tendency from yellow to green. Even at a low injection of 10 mA, the luminous color of sample h appeared to be more cyan. As the ratio of the current to the ITO area increases, i.e., the smaller the ITO area, the more current can reach the (1-101) and (10-10) planes, resulting in the greenish-blue color emission. Moreover, the distribution of light emission is relatively uniform over the entire ITO area for all the NW LEDs. Normally, it is a challenge to conformally deposit ITO on the sidewall of the NWs, while the uniformity of ITO can be improved by growing the p-GaN shape with a small (0001) plane and a large (1-101) plane [47]. In this experiment, the slightly connected p-GaN shell structures are beneficial to improve the uniformity of the ITO layer on the entire NW structures, and lateral current is spreading in the NW LEDs even with a small ratio of p-electrode to ITO area.

To gain insight into the relationship between emission peaks and the ITO mesa area of the LEDs, the EL peaks of each sample were separated by the Gaussian fitting. Figure 5(g) and (h) plot the peak wavelengths of samples a, d, f, g, and h as a function of injection current density from 0 to 405 A/cm^2^ and 400–4000 A/cm^2^, respectively. From the Gaussian fitting, two or three peak wavelengths were derived for samples a, d, f, g, and h under different injection currents. The variation tendency of the emission peaks is consistent among the samples. Basically, the NWs are featured with a blueshift from the (0001) and (1-101) planes at the apex region to the (10-10) plane, as confirmed by cathodoluminescence measurement in our previous report [48]. As described in Figure 4(f), the current reached the bottom of the NW as the current increased in MQS NW LEDs. In Figure 5(g), the long wavelength components above 525 nm were mainly emitted from the (0001) plane region with low crystallinity, which was the dominant emission in samples a, d, f, and g. Under low current injection density, the emission exhibited a clear blueshift from 675 to 525 nm. Furthermore, the short wavelength components near 490 nm are relatively stable, dominated by the emission from the (1-101) planes. Regarding sample h, at the current densities above 1400 A/cm^2^, a stable emission peak located at approximately 457 nm was confirmed, which was associated with the emission from the nonpolar (10-10) plane. Overall, the peak wavelengths from the (1-101) semipolar and the (10-10) nonpolar planes appeared to be stable and irrelevant to the size of the ITO mesa area. The results suggest that it is the potential to realize high emission stability in NW-based micro-LEDs by suppressing the emission from the (0001) plane and promoting the current injection into the (1-101) and (10-10) planes.

**Figure 6:**
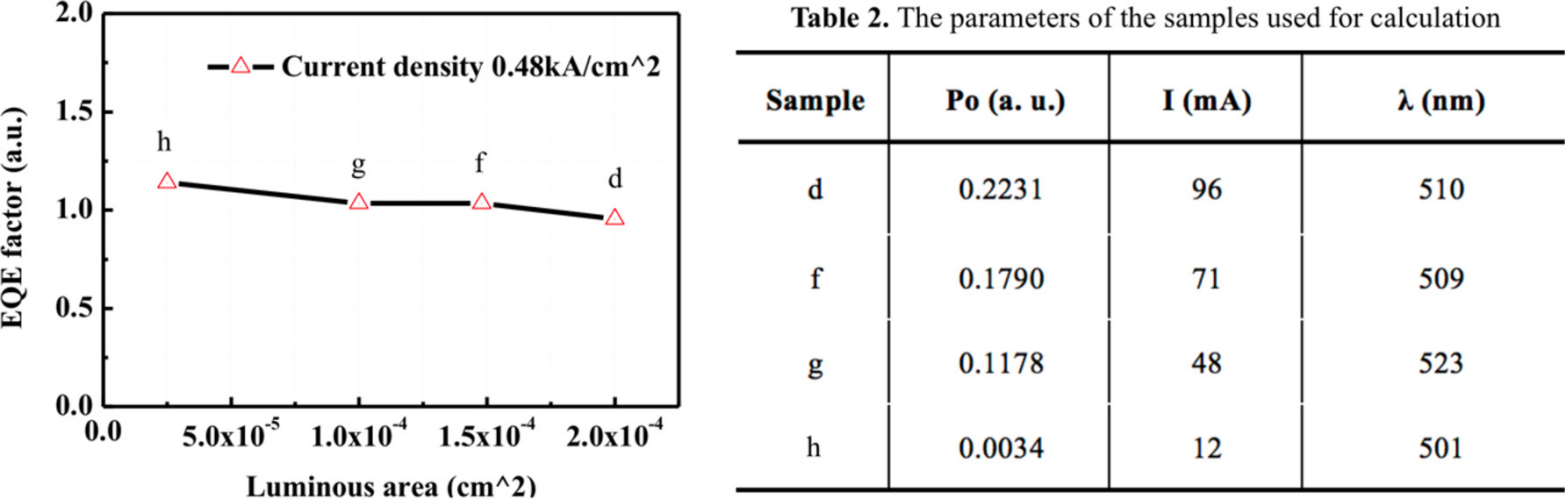
Estimated EQE factor as a function of the luminous area of the samples d, f, g, and h. The parameters of photons emitted per second (*P*_0_), current (*I*), and peak wavelength (*λ*) in Table 2 were derived from the measurement results.

Because the light output was detected from the backside and the emission light cannot be converted into an absolute value, an approximate EQE factor was derived by the formula: EQE factor [a.u.] = $\frac{P_{\text{0}}}{I}\times \frac{\lambda e}{hc}$ [49], where *h* is the Planck constant, *e* is the elementary electric charge, and *c* is the speed of light. The values of photons emitted per second (*P*_0_), injection current (*I*), and peak wavelength (*λ*) were determined at the current density of 0.48 kA/cm^2^. Although the practical current density of micro-LEDs is quite low, the high value of current density in this work was considered based on the varied current injection through entire NWs. The derivation of EQE factors at low current density is not sufficiently corresponding to the emission from the (1-101) and (10-10) planes of the NWs, because weak emission from the (0001) plane region was dominant, as confirmed in Figure 5(g) and (h). Therefore, the EQE factors were derived at a high current density, where emission from the (1-101) and (10-10) plane regions became prominent. Focusing on the characteristics of samples d, f, g, and h with different mesa areas, the EQE factors at the same current density are plotted in Figure 6. The parameters for EQE factor calculation are summarized in Table 2, as shown on the right of Figure 6. Since the measurements were taken under identical conditions, the light outputs and calculated EQE factor are comparable. It can be confirmed that degradation of the EQE factor did not occur as the mesa area was reduced. This is attributable to the feeblish influence of non-radiative recombination from a few etched NWs. Besides, the larger the mesa area, the more likely it is that dysfunctional NWs are contained. By applying an approach to suppress emission from the (0001) plane region [46, 48], it is expected to achieve NW-based micro-LEDs without obvious EQE degradation even at low current density. Therefore, MQS NWs have a distinct possibility for realizing efficient micro-LED owing to the minimized exposed area of the active region after mesa etching, which can reduce the surface recombination centers near the edge of the mesa.

### 3.3 Effect of different p-electrode areas on the optical characteristics

**Figure 7:**
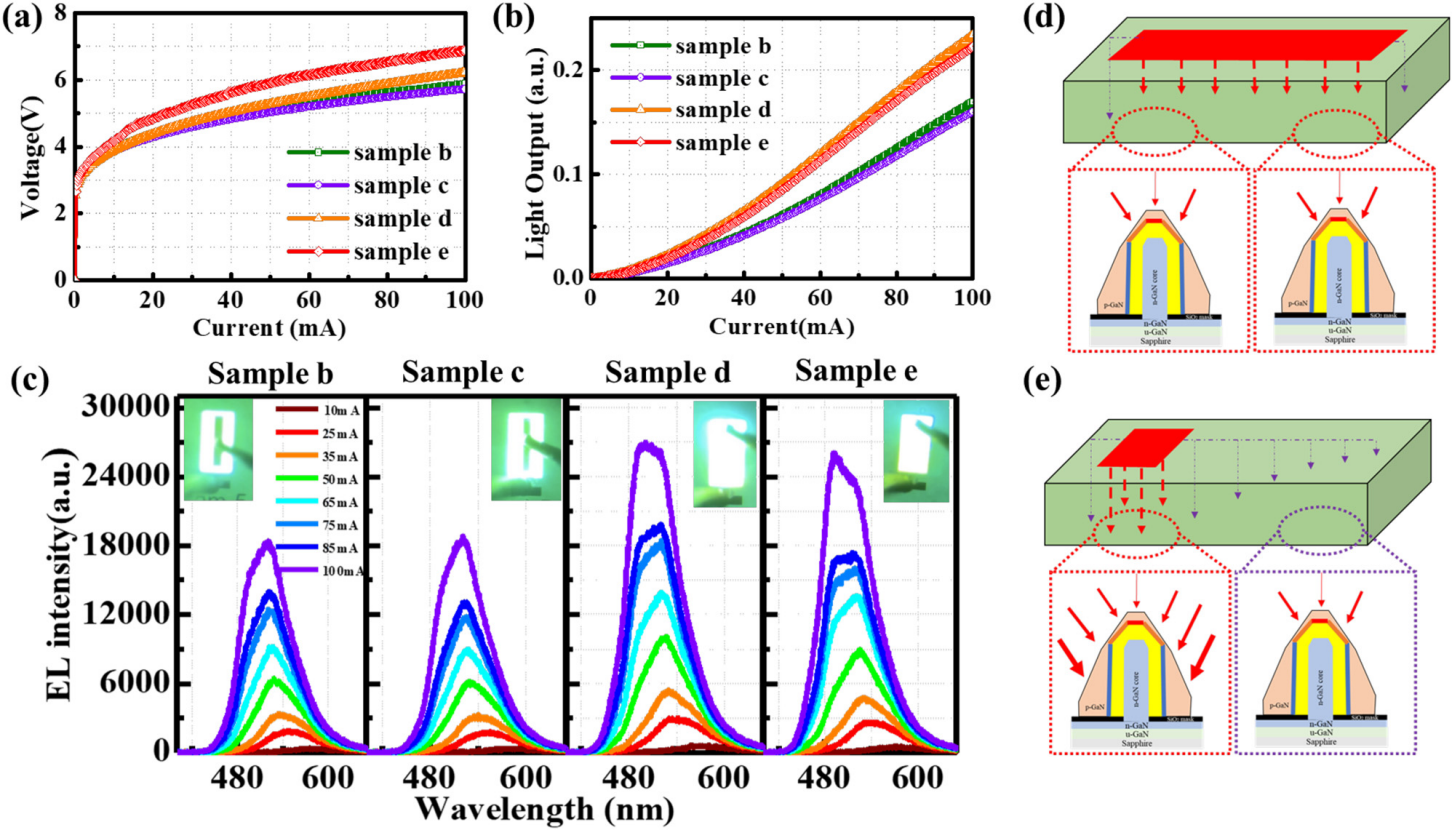
*I*–*V*–*L* characteristics, EL spectra, and corresponding current injection paths of the samples with different types of p-electrodes. (a) *I*–*V* and (b) *I*–*L* characteristics of the samples b, c, d, and e with different p-electrode areas. (c) EL spectra of the samples b, c, d, and e under different current injections. The insets show the emission photograph at the injection current of 100 mA. Schematic diagrams of current spreading in (d) samples b, c and (e) samples d, e.

The luminous photos in the above section demonstrated the uniform emission in the entire mesa region in the LED, even with low injection currents. The effect of different p-electrode area on the emission properties was investigated to further confirm the current diffusion in the NW LEDs. Samples b, c, d, and e were fabricated with the same ITO area of 200 × 100 μm^2^ and different p-electrode area of 190 × 40, 180 × 30, 40 × 40, and 30 × 30 µm^2^, respectively. As shown in the *I*–*V* curves in Figure 7(a), a higher the operation voltage was observed in sample e with a smaller p-electrode area due to the limited current diffusion through the p-electrode. However, the light output is higher in samples d and e than that in samples b and c, as shown in Figure 7(b). The slope at high current injections above 40 mA was increased in samples d and e, which implies that the light-emitting plane in NWs changed. The light was mainly emitted from the apex region of under low current injection, and then the current was injected into the (1-101) plane and (10-10) plane as the current increased. Figure 7(c) shows that the EL intensity was relatively higher in the samples d and e with small electrode areas, and two prominent emission peaks were distinguished at high injection currents. Comparing the inserted emission photographs, samples b and c are dominated by green light emission over the entire surface, while brighter blue color was observed around the electrodes of samples d and e. As illustrated in Figure 7(d), if the p-electrode area is large, the current can be injected uniformly over the entire chip. However, the injected current was mainly localized in the top region of the NWs, resulting in a relatively longer emission wavelength and weaker emission intensity in samples b and c. In samples d and e, with a smaller p-electrode area located at one side of the mesa region, the current diffusion gradually decreases through the device, as shown in Figure 7(e) schematic diagram. Under the same current injection, it is considered that the small p-electrode in samples d and e increased the light emission from the (1-101) and (10-10) planes of the NWs underneath the p-electrode, which contributed to the increased emission intensity of the short wavelength component. Therefore, the thicker p-GaN shell at the bottom area of the NWs also resulted in a slightly higher operation voltage in samples d and e because the current paths were concentrated around the p-electrodes. From these results, it can be seen that there is a trade-off between the current diffusion through all the NWs and effective injection to the sidewall of each NW in the LED devices, and a further optimization is required to combine these two aspects in the micro-LEDs.

## 4 Conclusions

In this work, we investigated the effect of the mesa and p-electrode areas on the current injection path in coaxial GaInN/GaN MQS NW LED. As the ITO mesa area became smaller, the current density increased. The current reached the lower part of the MQS NWs, which increased the EL intensity with dominant emission in the short wavelength region. From the Gaussian fitting of the EL spectra, the peak wavelengths from the (1-101) semipolar and the (10-10) nonpolar planes appeared to be stable and irrelevant to the size of the ITO mesa area. Nevertheless, the reverse leakage current in the NW LED was linearly increased as a function of the mesa size because the leakage path is mainly associated with the defective MQS structure on the (0001) plane area of each NW. The EQE factors derived from the light outputs, suggested that the stable emission efficiency is attributable to the possible influence of non-radiative recombination from a few etched NWs. Under the same current injection, it is considered that the small p-electrode area increased the light emission from the (1-101) and (10-10) planes of the NWs underneath the p-electrode, which contributed to the increased emission intensity of the short wavelength component. From these results, it can be seen that there is a trade-off between the current diffusion through all the NWs and effective injection to the sidewall of each NW in the LED devices. Therefore, MQS NWs have a high possibility for realizing efficient micro-LED through suppressing light emission from the (0001)-plane region and optimizing the dimension of the p-electrode area for current injection.
